# Digital technologies in primary care: Implications for patient care and future research

**DOI:** 10.1080/13814788.2022.2052041

**Published:** 2022-07-11

**Authors:** Ana Luísa Neves, Jako Burgers

**Affiliations:** a NIHR Imperial Patient Safety Translational Research Centre, Imperial College London, London, UK; b Centre for Health Technology and Services Research/Department of Community Medicine, Information and Decision in Health, Faculty of Medicine, University of Porto, Porto, Portugal; c Department Family Medicine, Care and Public Health Research Institute, Maastricht, The Netherlands; d Dutch College of General Practitioners, Utrecht, The Netherlands

**Keywords:** Telemedicine, primary care, quality of care, research methodology

## Abstract

Digital health is the convergence of digital technologies with health, healthcare, living, and society. Contrasting with the slow trend during the last decades, in the last few years, we have observed an expansion and widespread adoption and implementation. In this paper, we revisit the potential that digital health presents for the delivery of higher quality, safer and more equitable care. Focussing on three examples – patient access to health records, big data analytics, and virtual care – we discuss the emerging opportunities and challenges of digital health, and how they can change primary care. We also reflect on the implications for research to evaluate digital interventions: the need to evaluate clear outcomes in light of the six dimensions of quality of care (patient-centredness, efficiency, effectiveness, safety, timeliness, and equity); to define clear populations to understand what works and for which patients; and to involve different stakeholders in the formulation and evaluation of the research questions. Finally, we share five wishes for the future of digital care in General Practice: the involvement of primary healthcare professionals and patients in the design and maintenance of digital solutions; improving infrastructure, support, and training; development of clear regulations and best practice standards; ensuring patient safety and privacy; and working towards more equitable digital solutions, that leave no one behind.

KEY MESSAGES

- During the COVID-19 pandemic, digital health technologies have been adopted and implemented unprecedentedly.
- Digital health presents a potential for the delivery of higher quality, safer, and more equitable care, including primary care.
- There are opportunities and challenges regarding patient access to health records, big data analytics, and virtual care primary care.

## Introduction

Digital health is the convergence of digital technologies with health, healthcare, living, and society, aiming to deliver high quality care [1–3]. This growing discipline capitalises on information and communication technologies, including electronic health records, health analytics and data visualisation; wearables and mobile health, telemedicine and remote care, among others [4,5]. Offering an opportunity to deliver holistic, patient-centred, and tailored care, digital health emerges as a promising opportunity to tackle many of the challenges of primary care [6]. However, implementation has been slow [7,8]. With the COVID-19 pandemic, the landscape has changed abruptly, and patients, providers and systems were forced to embrace digital technologies as an integral part of healthcare. In this piece, we focus on three applications of digital health: patient access to health records, big data analytics, and virtual care. For each of them, we discuss the emerging opportunities and challenges of digital health, how they have evolved and their potential to shape the future of General Practice.

## Patient access to health records

Sharing health information with patients can improve patient engagement, care quality, and, consequently, health outcomes [9]. In a recent review, we evaluated 20 years of published evidence assessing the impact of sharing electronic health records with patients on several aspects of quality of care [10]. Inconsistent results were found for patient-centredness outcomes (including satisfaction, activation, self-efficacy, empowerment, and health literacy). However, it is important to note that half of the studies demonstrated a beneficial effect. Concerning efficiency, most studies found either a reduction in healthcare usage or no change. A beneficial effect was observed in various safety outcomes, including general adherence and medication safety. Importantly, meta-analyses showed a beneficial effect in effectiveness, highlighting a reduction in the absolute values of HbA1c in patients with type 2 diabetes, which remained significant in sensitivity analyses for low risk of bias studies and long-term interventions only. Importantly, no studies focussed specifically on the impact on timeliness or equity.

As evidence increasingly supports that patient records belong to patients and should be easily available in a structured, electronic form, new questions arise [11]. Given the complexity of the health information provided, access per se is unlikely to improve current gaps in care or significantly improve morbidity or mortality. Therefore, training (rather than access alone) should be an integral part of implementation strategies. Equally, special attention must be paid to disparities in access and use of electronic health records by low-income, racial and ethnic minorities, lower literate and linguistically diverse populations [12]. Recently, we have evaluated the determinants of usage of the Care Information Exchange (the largest shared patient portal programme in the UK, hosting the records of over 2.3 million people living in North West London) and observed that individuals with a higher educational degree or higher digital literacy scores had higher odds of being a portal user [13]. It is critical that further research systematically addresses these inequalities through patient-centred interventions aiming to reduce the digital divide and engage effectively with underserved or excluded groups of patients.

## Big data analytics

Electronic health records were primarily developed to improve individual care. However, with the advent of novel data mining techniques and increased computational power, healthcare services realised the potential of using healthcare non-identified data for broader purposes. These purposes include, for example, using sizeable primary care datasets to understand different profiles of patients. For instance, analysing how different groups of patients use the healthcare system (i.e. number and type of primary care and secondary care visits) may help us to understand their service needs and care priorities, identify low- and high-need groups, and tailor interventions for different user types [14]. Big data analytics can also help us understand differences between geographical areas, for instance, to understand the local prevalence of chronic diseases (e.g. diabetes, hypertension, chronic pulmonary obstructive disease) and consequently inform the allocation of human resources to ensure adequate follow-up and management. In addition, big data analytics can support quality improvement in general practice: collecting and monitoring data on structural elements (i.e. characteristics of the community, institution, provider and patient), processes of care (i.e. treatment appropriateness and services provided to specific groups) and, ultimately, health outcomes.

Big data in healthcare is also a powerful resource for research purposes. In the evaluation of drug effectiveness, for instance, primary care has heavily relied on randomised trials performed in inpatient and secondary care settings [15]. Those often included particular and non-diverse groups of patients that do not represent the typical patient presenting in general practice [15]. Using electronic health records for these purposes opens new avenues to explore real-life data to inform clinical decisions on diagnosis, management, and follow-up in the primary care setting, in addition to the evidence from randomised controlled trials [16].

Finally, it is essential to reflect on data ownership. Whenever we discuss secondary uses of electronic health records (i.e. approaches that go beyond direct patient benefit), general practitioners should not forget that patients are ultimately the owners of the data, and their views and concerns about data sharing for secondary purposes must be adequately considered. In a recent study, we used an immersive exhibition to invite the public to reflect on their main health record data sharing concerns. While participants reported hopes around increased interoperability and collaboration, generation of evidence for higher quality and safer care, and delivery of more personalised care, they also expressed fears concerning inadequate security, data inaccuracy, discrimination and inequality [17]. This urges primary care professionals to be aware of the ethical implications of sharing data and their responsibilities [18].

## Virtual care

Virtual care encompasses all methods used to communicate and interact with patients remotely. Initially, it emerged as a solution to bring care to remote areas, where access to care had proven to be particularly difficult. We have witnessed a growing interest in virtual care in both high- and low-income primary care settings in recent decades. Many health care systems have taken substantive steps to embed virtual care initiatives into primary care, including the National Health Service in England and the Kaiser Permanente in the United States. All over the world, many other healthcare systems have equally advocated for the adoption of virtual solutions to complement face-to-face care [19–25]. But despite the promise, the widespread adoption of virtual primary care has grown slowly, limited by cultural, regulatory, technical and financial barriers [19,26,27].

However, in the last year, virtual care approaches have taken centre stage: triaging and monitoring COVID-19 patients and other acute conditions in primary care, but also ensuring access and continuity of care for patients with long-term conditions (e.g. diabetes, hypertension, asthma, psychiatric illnesses, chronic pain) [22–24]. Whilst most of the previous evidence on virtual primary care came from small studies, the COVID-19 pandemic forced all stakeholders (patients, healthcare providers, and healthcare systems) to embrace virtual consultations as their main route of care. As difficult as it might be to find silver linings in the context of a global crisis, this unprecedented adoption without robust evidence of effectiveness presents a unique opportunity to learn more about the main challenges and benefits experienced and incorporate these lessons into the future of primary care [28,29]. Therefore, it is critical to now evaluate the perceived impact on quality and safety of care through well-designed randomised controlled trials comparing different approaches of virtual care with usual (non-virtual) care to determine the essential factors for high-quality, sustainable use of virtual care in the future [30].

## Implications for research in primary care

With the increasing use of digital solutions, there is a growing need to evaluate their impact on primary care, including risks and benefits, and to inform health policies that are both patient-centred and evidence-based. As research on digital care covers the whole of medicine, including clinical and contextual issues, defining the focus of research is essential for addressing the most important issues faced by patients and society. A research agenda independent from commercial interests and preferences of funding agencies could help focus on issues that matter most. All relevant stakeholders in primary care should be able to contribute in defining and selecting research questions to reduce any bias. Public and patient involvement is needed to capture diversity and ethical issues. The Dutch general practice research agenda could be seen as an example, also including e-Health as a theme [31].

In formulating research questions, the patient population should be well-described. The population in primary care is often heterogeneous and characterised by differences in age, gender, ethnicity, education, and health status. The availability of internet and mobile devices can also largely differ. These features should be considered in analysing the effectiveness of digital technologies. The content and how to use the digital application should be apparent, anticipating implementation and widespread use. In assessing the use and effectiveness of digital care, different outcome measures could be defined. These could include clinical or health outcomes, consultation, perceived quality of care, and patient and professional satisfaction. Positive and negative effects can vary among different outcomes. One may argue whether digital care should improve the quality of care compared to usual care or should it be non-inferior, which will affect the study design.

When developing research questions to evaluate the impact of digital health interventions, it might be helpful to consider the six dimensions of quality of care, as defined by the Institute of Medicine – these include safety, effectiveness, patient-centredness, timeliness, efficiency, and equity (Box 1) [32]. Safety may be a critical aspect, following Hippocrates’ oath to ‘first avoid harm’ to patients. Digital care could be risky in specific conditions that still needs face-to-face contact, such as acute care and palliative care. Effectiveness of care is central in assessing the benefits and added value of digital care. Providing person-centred care is eminently related to digital care as individual preferences, needs, and values should always be considered in decision making on digital applications. Timeliness must be addressed in light of the opportunities to reduce time-to-diagnosis and time-to-treatment while reducing waiting times for consultations and referrals. Efficiency or cost-effectiveness is critical from an organisational and policy perspective. There might be important benefits, including reducing personnel, equipment, travel costs, and supplies, but there are also non-negligible costs associated with set-up and maintenance of digital technologies. Last but not least, the aim is to provide equitable care. This may be difficult as the availability and costs of digital technology, such as mobile devices for patients, is not covered by all healthcare insurers and plans. Moreover, digital care demands complex skills that may vary among the patient population, such as low literacy or limited digital skills, and these differences may entrench existing inequities in access to and use of health care [33,34]. Future research should focus on the wider measurement of patient interest, access and skills to using technology-based health platforms and tools, and interventions should be tailored to match patient preferences and needs [14]. The use of mixed-method and implementation science studies may help evaluate clinical impact and use, usability, and uptake of digital technologies [14,35].

Box 1 Aims for health care improvement (Institute of Medicine, 2001) [28].

- Safety: Avoiding harm to patients from the care that is intended to help them.
- Effectiveness: Providing services based on scientific knowledge to all who could benefit and refraining from providing services to those not likely to benefit (avoiding underuse and misuse, respectively).
- Patient-centredness: Providing care that is respectful of and responsive to individual patient preferences, needs, and values and ensuring that patient values guide all clinical decisions.
- Timeliness: Reducing waits and sometimes harmful delays for both those who receive and those who give care.
- Efficiency: Avoiding waste, including waste of equipment, supplies, ideas, and energy.
- Equity: Providing care that does not vary in quality because of personal characteristics such as gender, ethnicity, geographic location, and socioeconomic status.

## The way forward: five wishes for the future of digital care

The increasing use of digital care challenges general practice in changing the expectations of patients and society, and posing new challenges to research in primary care. Specific expertise is needed to understand and oversee new developments and technologies while we refine digital innovation as part of our learning journey. In this context, we have five wishes for the future of digital care (Box 2). First, primary healthcare professionals, as well as patients, should be involved in the design and maintenance of digital solutions. Second, infrastructure, support, and training should be prioritised, enhancing awareness and skills of both patients and providers. In this context, there is a need to improve the infrastructure supporting data reuse [36]. Adhering to a set of principles (Findability, Accessibility, Interoperability, and Reusability [FAIR data]) can maximise the added-value gained; this applied not only to ‘data’ in the conventional sense, but equally to the algorithms, tools, and workflows that generate data [36]. Third, data sharing must be enhanced, and clear regulations and best practice standards adopted to minimise variations in the quality of the solutions offered. This may include developing digital health certification standards that support person-centred care, enhance user experience, ensure equitable access, and hold vendors accountable [37]. National agencies and professional societies could actively contribute to setting quality standards. Fourth, patient safety and privacy should be guaranteed. Patients are owners of their health data and need ensure that their data are not misused for other purposes or shared without their informed consent. Fifth, digital care should not aggravate existing health inequities. Collaboration between all stakeholders in healthcare – professionals, policymakers, patients, and industry – could help realising this ultimate goal. The COVID-19 pandemic has shown the need and relevance of collaboration as part of a global community; it is now time to develop a shared agenda that supports collaboration in general practice, research and policy and facilitates the delivery of digital solutions that leave no one behind.

Box 2 Five wishes for the future of digital care.

1. Co-design with primary healthcare professionals and patients
2. Better infrastructure, support & training
3. Data sharing, clear regulations & best practice standards
4. Ensure patient safety & privacy
5. Care that leaves no one behind
